# DSNN: A DenseNet-Based SNN for Explainable Brain Disease Classification

**DOI:** 10.3389/fnsys.2022.838822

**Published:** 2022-05-26

**Authors:** Ziquan Zhu, Siyuan Lu, Shui-Hua Wang, Juan Manuel Gorriz, Yu-Dong Zhang

**Affiliations:** ^1^School of Computing and Mathematical Sciences, University of Leicester, East Midlands, United Kingdom; ^2^School of Computer Science and Technology, Henan Polytechnic University, Jiaozuo, China; ^3^Department of Signal Theory, Networking and Communications, University of Granada, Granada, Spain; ^4^Guangxi Key Laboratory of Trusted Software, Guilin University of Electronic Technology, Guilin, China

**Keywords:** brain diseases, convolutional neural network, randomized neural network, DenseNet, MRI

## Abstract

**Aims**: Brain diseases refer to intracranial tissue and organ inflammation, vascular diseases, tumors, degeneration, malformations, genetic diseases, immune diseases, nutritional and metabolic diseases, poisoning, trauma, parasitic diseases, etc. Taking Alzheimer’s disease (AD) as an example, the number of patients dramatically increases in developed countries. By 2025, the number of elderly patients with AD aged 65 and over will reach 7.1 million, an increase of nearly 29% over the 5.5 million patients of the same age in 2018. Unless medical breakthroughs are made, AD patients may increase from 5.5 million to 13.8 million by 2050, almost three times the original. Researchers have focused on developing complex machine learning (ML) algorithms, i.e., convolutional neural networks (CNNs), containing millions of parameters. However, CNN models need many training samples. A small number of training samples in CNN models may lead to overfitting problems. With the continuous research of CNN, other networks have been proposed, such as randomized neural networks (RNNs). Schmidt neural network (SNN), random vector functional link (RVFL), and extreme learning machine (ELM) are three types of RNNs.

**Methods**: We propose three novel models to classify brain diseases to cope with these problems. The proposed models are DenseNet-based SNN (DSNN), DenseNet-based RVFL (DRVFL), and DenseNet-based ELM (DELM). The backbone of the three proposed models is the pre-trained “customize” DenseNet. The modified DenseNet is fine-tuned on the empirical dataset. Finally, the last five layers of the fine-tuned DenseNet are substituted by SNN, ELM, and RVFL, respectively.

**Results**: Overall, the DSNN gets the best performance among the three proposed models in classification performance. We evaluate the proposed DSNN by five-fold cross-validation. The accuracy, sensitivity, specificity, precision, and F1-score of the proposed DSNN on the test set are 98.46% ± 2.05%, 100.00% ± 0.00%, 85.00% ± 20.00%, 98.36% ± 2.17%, and 99.16% ± 1.11%, respectively. The proposed DSNN is compared with restricted DenseNet, spiking neural network, and other state-of-the-art methods. Finally, our model obtains the best results among all models.

**Conclusions**: DSNN is an effective model for classifying brain diseases.

## Introduction

Brain diseases refer to intracranial tissue and organ inflammation, vascular diseases, tumors, degeneration, malformations, genetic diseases, immune diseases, nutritional and metabolic diseases, poisoning, trauma, parasitic diseases, etc. Brain diseases often show disorders of consciousness, sensation, movement, or autonomic nerve dysfunction. There may also be fever, headache, vomiting, and other mental symptoms. Taking Alzheimer’s disease (AD) as an example, the number of patients dramatically increases in developed countries. By 2025, the number of elderly patients with AD aged 65 and over will reach 7.1 million, increasing nearly 29% over the 5.5 million patients of the same age in 2018 (Lynch, 2018). Unless medical breakthroughs are made, the number of Alzheimer’s patients aged 65 and over may increase from 5.5 million to 13.8 million by 2050, almost three times the original.

Now, brain diseases are mainly diagnosed by doctors. However, the manual diagnosis requires much time. At the same time, different doctors may have different views on the same examination results, which has brought a lot of trouble to patients.

More and more researchers use computational methods (Wang et al., 2021) to classify brain diseases. Noreen et al. (2020) introduced a multi-level method using two DensNet201 and Inception-v3 to diagnose early brain tumors. Finally, the accuracy of Inception-v3 and DensNet201 were 99.34% and 99.51%, respectively. Amin et al. (2019b) presented a model using magnetic resonance images to automatically classify brain tumors according to the LSTM model method. What’s more, this method obtained 0.97 DSC in practical application. Amin et al. (2019a) used a deep learning model to predict healthy and unhealthy brain tumor slices. Arunkumar et al. (2020) introduced a new model to identify ROI location based on brain tumor MRI. The method finally got 89% sensitivity, 92.14% accuracy, and 94% specificity. Purushottam Gumaste and Bairagi (2020) proposed an algorithm to extract left and right brain features. This article also introduced different statistical feature extraction methods and used a support vector machine to extract tumor regions from statistical features. Chatterjee and Das (2019) proposed a novel method for the segmentation of brain images, which were divided into two categories: benign (low level) and evil (high level). Bhanothu et al. (2020) presented a new method according to R-CNN to detect tumors and mark their location. Finally, the detection and classification accuracy of the three types of brain tumors were 89.45%, 68.18%, and 75.18%. Natekar et al. (2020) compared various technologies for brain tumor segmentation models and visualized the internal concepts to have a deeper understanding of how these technologies segmented with high accuracy. Aboelenein et al. (2020) introduced a novel network (HTTU-Net) for brain tumor cutting. Huang et al. (2020) presented the differential feature neural network (DFNN) method. The method introduced DFM blocks and combined SE blocks. When the DFM block was introduced, the accuracy of the two databases was improved by 1.8% and 1.3%, respectively. Hu and Razmjooy (2020) proposed a meta heuristic-based system to detect tumors. Sadad et al. (2021) introduced a novel model according to UNET architecture and ResNet50 as the backbone for the detection of brain tumors. Kalaiselvi et al. (2020) proposed a patch-based-updated run-length region growth (PR2G) method to detect and segment tumors. The accuracy of this method was 97%. Kaplan et al. (2020) used two methods to classify the three different types of brain tumors. The two methods were *n*LBP and αLBP. The highest classification accuracy of brain tumors was 95.56%. Khalil et al. (2020) proposed a new method (DA clustering) to improve the accuracy of extracting initial contour points to detect three-dimensional magnetic resonance brain tumors better. Khan et al. (2020) proposed a new method, partial tree (PART), to detect brain tumors of grade I to grade IV brain tumors. This method used the rule learner of an advanced feature set. Ma and Zhang (2021) proposed a method to intelligently detect brain tumors based on a lightweight neural network. Hollon et al. (2020) introduced a new method for the automatic detection of brain tumors by combining SRH 5–7, CNN, and the label-free optical imaging method. Saba et al. (2020) used a new method to detect brain tumors. The Grasp cut method was used to segment brain tumor symptoms, and VGG-19 was used to obtain features. Sharif et al. (2020) proposed an unsupervised fuzzy set method for brain tumor segmentation. The triangular fuzzy median filter enhanced the image to better detect brain tumors. Xu et al. (2020) presented a new structure for the early detection of brain tumors. The new structure was mainly composed of five parts: tumor segmentation, morphology, denoising, feature extraction, and classification. Hemanth et al. (2011) introduced a novel method (HSBPN) to segment MR brain tumor images. Nayef et al. (2013) introduced a novel structure for the classification of the MRI dataset. Chen et al. (2017) presented an improved method for detecting pathological brains. A new classifier was used in the improved method. Shoeibi et al. (2020) finished a review on the segmentation of the Covid-19 by DL. Shoeibi et al. (2021b) performed a comprehensive survey about the application of DL in the detection of multiple sclerosis. Sadeghi et al. (2021) showed a survey on the automatic diagnosis of the SZ by AI. Shoeibi et al. (2021a) completed a comprehensive review on the application of the various AI techniques in the diagnosis of epileptic seizures. Shoeibi et al. (2021c) completed a review of various methods based on DL for automatic diagnosis of SZ by electroencephalogram (EEG) signals. Shoeibi et al. (2022) proposed a new model to automatically detect Epileptic seizures. The proposed model was based on the DL and the fuzzy theory. Odusami et al. (2022) proposed a method for the recognition of AD. They tested two CNN models (DenseNet201 and ResNet18) to perform this task. This method obtained 98.86% accuracy, 98.94% precision, and 98.89% recall. Razzak et al. (2022) introduced a new network (PartialNet) to detect AD based on MRIs. This network achieved improvements on the AD detection. Ashraf et al. (2021) experimented with different CNN models to detect AD based on transfer learning. Finally, the fine-tuned DenseNet got the highest accuracy (99.05%).

If brain diseases are diagnosed manually, doctors need to spend a lot of time on examination. Sometimes we may encounter the problem that different doctors have different views on the examination results of the same patient. As shown in Table 1, most researchers use deep convolution neural networks (DCNNs) to classify and identify brain diseases. However, there will be many parameters and calculations in the training of DCNN, which can lead to a long training time (Zhang et al., 2021). At the same time, DCNNs need a sea number of experimental data for training because a small number of experimental data may lead to overfitting problems (Górriz et al., 2020; Zhang et al., 2020).

**Table 1:** Contributions of state-of-the-art methods.

Method	Contribution
Noreen et al. (2020)	A multi-level method using two DensNet201 and Inception-v3 was proposed to diagnose early brain tumors.
Amin et al. (2019b)	A model according to the LSTM model method using magnetic resonance images was introduced to classify brain tumors automatically.
Amin et al. (2019a)	A deep learning model was used to predict healthy and unhealthy brain tumor slices.
Arunkumar et al. (2020)	A new model was introduced to train MRI brain tumors to identify ROI location.
Purushottam Gumaste and Bairagi (2020)	An algorithm was proposed to extract left and right brain features. This article also introduced different statistical feature extraction methods and used a Support Vector Machine to extract tumor regions from statistical features.
Chatterjee and Das (2019)	A novel method was proposed for the segmentation of brain images.
Bhanothu et al. (2020)	A new method based on R-CNN was presented to detect tumors and mark their location.
Natekar et al. (2020)	Various technologies were compared for brain tumor segmentation models.
Aboelenein et al. (2020)	The HTTU-Net was proposed for brain tumor cutting.
Huang et al. (2020)	The DFNN was proposed. The method introduced DFM blocks and combined SE blocks.
Hu and Razmjooy (2020)	A meta heuristic-based system was presented to detect tumors.
Sadad et al. (2021)	A novel model according to UNET architecture and ResNet50 as the backbone was proposed for the detection of brain tumors.
Kalaiselvi et al. (2020)	The PR2G was proposed to detect and segment tumors.
Kaplan et al. (2020)	Then LBP and αLBP were used to classify the three different types of brain tumors.
Khalil et al. (2020)	The DA clustering was proposed to improve the accuracy of extracting initial contour points to detect three-dimensional magnetic resonance brain tumors better.
Khan et al. (2020)	The PART was introduced to detect brain tumors of grade I to grade IV brain tumors.
Ma and Zhang (2021)	A method was proposed to intelligently detect brain tumors based on a lightweight neural network.
Hollon et al. (2020)	A new method was proposed for the automatic detection of brain tumors by combining SRH 5–7, CNN, and the label-free optical imaging method.
Saba et al. (2020)	A new method was proposed to detect brain tumors. The Grasp cut method was used to segment brain tumor symptoms, and VGG-19 was used to obtain features.
Sharif et al. (2020)	An unsupervised fuzzy set method was introduced for brain tumor segmentation.
Xu et al. (2020)	A new structure was proposed for the early detection of brain tumors. The new structure was mainly composed of five parts: tumor segmentation, morphology, denoising, feature extraction, and classification.
Hemanth et al. (2011)	The HSBPN was proposed to segment MR brain tumor images.
Nayef et al. (2013)	A novel structure was presented for the classification of the MRI dataset.
Chen et al. (2017)	An improved method was introduced for detecting pathological brains.
Shoeibi et al. (2020)	A review was presented on the segmentation of the Covid-19 by DL.
Shoeibi et al. (2021b)	A comprehensive survey about the application of DL in the detection of Multiple Sclerosis
Sadeghi et al. (2021)	A survey was presented on the automatic diagnosis of the SZ by AI.
Shoeibi et al. (2021a)	A comprehensive review was presented on applying the various AI techniques in the diagnosis of Epileptic seizures.
Shoeibi et al. (2021c)	A review of various methods based on DL for automatic diagnosis of SZ by electroencephalogram (EEG) signals was completed.
Shoeibi et al. (2022)	A new model was proposed to detect Epileptic seizures automatically. The proposed model was based on the DL and the fuzzy theory.
Odusami et al. (2022)	A method was proposed for the recognition of AD. They tested two CNN models (DenseNet201 and ResNet18) to perform this task.
Razzak et al. (2022)	A new network (PartialNet) was introduced to detect AD based on MRIs. This network achieved improvements in AD detection.
Ashraf et al. (2021)	Different CNN models were experimented with to detect AD based on transfer learning. Finally, the fine-tuned DenseNet got the highest accuracy (99.05%).

To cope with the problems mentioned above, we propose three novel models to classify brain diseases automatically. They are: DenseNet-based Schmidt neural network (DSNN), DenseNet-based random vector functional link (DRVFL), and DenseNet-based extreme learning machine (DELM). We select DenseNet to extract features and use randomized neural networks (RNNs) for classification.

We modify the pre-trained DenseNet. Then, the modified DenseNet is fine-tuned on the dataset. In the DSNN, the last five layers within the fine-tuned DenseNet are substituted by the Schmidt neural network (SNN). In the DRVFL, we select the RVFL (RVFL) to substitute the last five layers of the fine-tuned DenseNet. In the DELM, we choose the extreme learning machine (ELM) to replace the end five layers of the fine-tuned DenseNet.Five-fold cross-validation is used to evaluate the proposed three models: DSNN, DRVFL, and DELM, in terms of aspects (Acc, Sen, Spe, Pre, and F1). We finally get thatDSNN gives the best performance among the three proposed models and overperforms the other six state-of-the-art algorithms. The five main innovations of this study are:

(1) DenseNet is validated as the backbone by experiments showing its superiority to AlexNet, ResNet-18, ResNet-50, and VGG.
(2) DSNN, DRVFL, and DELM are proposed by replacing the last five layers within the fine-tuned DenseNet with three randomized neural networks.
(3) The DSNN gets the best performance among the three proposed models.
(4) The DSNN overperforms the restricted DenseNet and spiking neural network by experiments.
(5) The DSNN is compared with six state-of-the-art algorithms and obtains the best results among the list methods.

The rest of this article is as follows. The dataset is given in Section “Materials". Section “Methodology" discusses the methodology. Section “Results and Discussion" is about the experiment results. We conclude this article in Section “Conclusion".

## Materials

**Figure 1:**
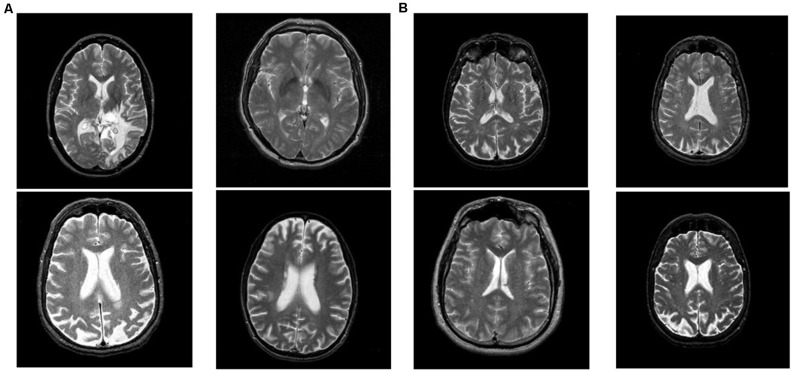
**(A)**Unhealthy and **(B)** healthy brain images in the dataset.

The dataset is downloaded from the Harvard Medical School website (Johnson and Becker, 2021). There are four types of brain diseases: cerebrovascular disease, neoplastic disease, degenerative disease, and inflammatory or infectious disease. This article classifies all four brain disease images as unhealthy brain images. A total of 177 unhealthy brain images and 20 healthy brain images are used in this article. The size of all images in this article is 256 × 256. Some unhealthy and healthy brain images in this article are shown in Figure 1. The left four images are the unhealthy brain images, and the right four are the healthy images.

## Methodology

### Proposed DSNN

Tables s 2, 3 give the acronym definitions and parameter definitions, respectively. More and more researchers devote energy to researching image classification technology (Lu et al., 2021). In image classification, feature extraction is a crucial step. However, the image contains too much messy information, so extracting valuable features is difficult. Decades ago, people usually manually extracted features. However, manual feature extraction takes much time, and the results are usually not ideal. With the continuous progress of computer technology, more and more people use computer models for image feature extraction (Leming et al., 2020). Many computer models are successful (Lu S. Y. et al., 2021), such as CNN models. The convolution layer in the CNN model can significantly reduce the volume of parameters to shorten the training time. Researchers have proposed many great CNN models, such as AlexNet (Lu et al., 2020a), MobileNet (Lu et al., 2020), ResNet (Lu et al., 2020b), and so on. This article proposes three models for the automatic classification of brain diseases: DSNN, DRVFL, and DELM. The DSNN gets the best performance among the three proposed models.

**Table 2:** Acronym and full explanation.

Acronym	Full explanation
AD	Alzheimer’s disease
Acc	Accuracy
Avr	Average
BN	Batch normalization
CNN	Convolution neural network
DCNN	Deep convolution neural network
DELM	DenseNet-based extreme learning machine
DL	Deep learning
DRVFL	DenseNet-based random vector functional link
DSNN	DenseNet-based Schmidt neural network
ELM	Extreme learning machine
F1	F1-score
FC	Fully connected
ML	Machine learning
Pre	Precision
RVFL	Random vector functional link
RNNs	Randomized neural networks
Sen	Sensitivity
SNN	Schmidt neural network
Spe	Specificity
Std	Standard deviation

**Table 3:** The definition of the parameter.

Parameter	Definition
*O*_*m*_	The output of the *M*-th layer
*T*_*m*_	The nonlinear transformation
*(**x_i_, y_i_**)*	The given dataset
*n*	The input dimension
*M*	The output dimension
***w_j_***	The weights vector
*d*_*j*_	The bias of the *j*-th hidden node
*P*	The final output weights
*q*	The output biases of SNN
**Y= (*y_1_,....,y_N_*)^T^**	The ground-truth label matrix of the dataset
**X= (*x_1_,....,x_N_*)^T^**	The input matrix
*s*()	The sigmoid function
*V*	The number of hidden nodes
**A**	The output matrix of the hidden layer

**Figure 2:**
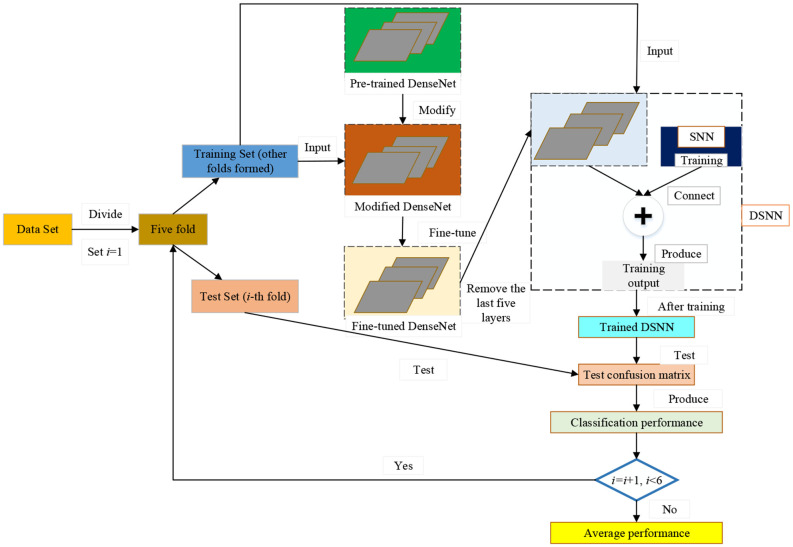
The pipeline of the proposed DSNN.

The pseudocode of the proposed DSNN is shown in Table 4. The pipeline of our model is given in Figure 2. We choose the pre-trained DenseNet as the backbone of the proposed DSNN. We modify the pre-trained DenseNet. Then, the modified DenseNet is fine-tuned on the dataset. The last five layers within the fine-tuned DenseNet are substituted by the Schmidt neural network (SNN). In our model, the fine-tuned DenseNet plays the role of feature extraction. The SNN is trained by the extracted features F from the fine-tuned DenseNet. Five-fold cross-validation is used to evaluate the proposed DSNN.

**Table 4:** Pseudocode of the proposed DSNN.

Step 1: Load the pre-trained DenseNet.
Step 2: Modify the pre-trained DenseNet.
Step 2.1 Remove softmax and classification layer from the pre-trainedDenseNet.
Step 2.2 Add FC128, ReLU, BN, FC2, softmax, and classification layer.
Step 3: Divide the dataset into five groups of the same size and set *i*=1
Step 4: Use the *i*-th group as the test set, and all the other groups form the training set.
Step 5: Fine-tune the modified DenseNet.
Step 5.1: Input is the training set.
Step 5.2: Target is the corresponding label.
Step 6: Replace the last five layers of the fine-tuned DenseNet with SNN.
Step 7: Extract features *F* as the output of the FC128 layer.
Step 8: Train the classifier of the DSNN on the extracted features *F* and the labels.
Step 8.1: Input is the extracted features.
Step 8.2: The target is the label of the training set.
Step 8.3: SNN is the classifier of the DSNN.
Step 9: Test the trained DSNN on the test set.
Step 10: Report the test classification performance of the trained DSNN.
Step 11: Set *i*= *i* + 1, if *i* < 6, go to Step 4.
Step 12: Average test classification performance.

### Backbone of the Proposed DSNN

The CNN models (Albawi et al., 2017) have been researched continuously in recent decades. In 1998, LeCun proposed LeNet (LeCun, 2015) with a five-layer structure. In 2014, the visual geometry group proposed VGG (Simonyan and Zisserman, 2014) with a 19-layer structure. The Highway Networks (Srivastava et al., 2015) were proposed later, with more than 100 layers.

**Figure 3:**
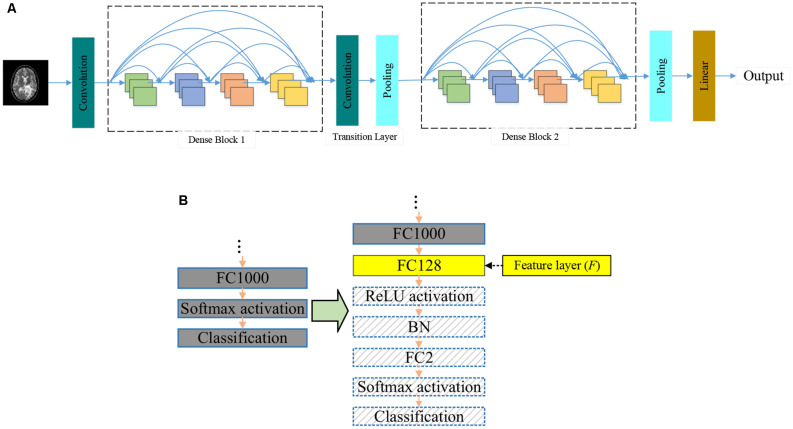
Backbone of the proposed DSNN. **(A)** The general view of DenseNet.**(B)** The modifications in the pre-trained DenseNet.

With the increasing number of network layers in CNN models, researchers are troubled by the problem of gradient vanishing. Batch normalization (BN) alleviates the problem of gradient vanishing to some extent. ResNet (He et al., 2016) reduces the gradient vanishing problem by constructing identity mapping. In 2017, DenseNet (Huang et al., 2017) was proposed to reduce the gradient vanishing problem by establishing dense connectivity between the front and rear layers. Dense connectivity makes more effective use of features than other networks. Thus, DenseNet can achieve better performance. The general view of DenseNet is given in Figure 3A.

Dense blocks refer to the specific blocks of DenseNet, as shown in Figure 3A. All the front layers are connected with the rear layers. In the same dense block, the height and width of each feature map will not change, but the number of channels will change. In the traditional sequential CNN, if you have *M* layers, there will be *M* connections, but DenseNet will introduce *M*(*M*+1)/2 more connections. Supposing there are *M* layers, *O*_M_ denotes the output of the *M*-th layer, *T*_M_ represents the nonlinear transformation. The comparison of DenseNet with other CNNs is listed below:

Traditional sequential CNN:


(1) $$O_{M}=T_{M}(O_{M-1})$$

ResNet:


(2) $$O_{M}=T_{M}(O_{M-1})+O_{M-1}$$

DenseNet:


(3) $$O_{M}=T_{M}[(O_{0},O_{1},...,O_{M-1})]$$

where [] is the concatenation.

The transition layer is a module that connects different dense blocks. Its primary function is to integrate the features obtained from the previous dense block and reduce its width and height.

Researchers used the ImageNet dataset to pre-train the DenseNet. There are 1,000 output nodes on the pre-trained DenseNet. However, this article only needs two output nodes. We modify the pre-trained DenseNet. The modifications are shown in Figure 3B.

After these modifications, we fine-tune the modified DenseNet by the training set. We remove the last five layers of the fine-tuned DenseNet and add SNN to improve the classification performance. In the proposed DSNN, the fine-tuned DenseNet is the feature extraction.

### Three Proposed Networks

**Figure 4:**
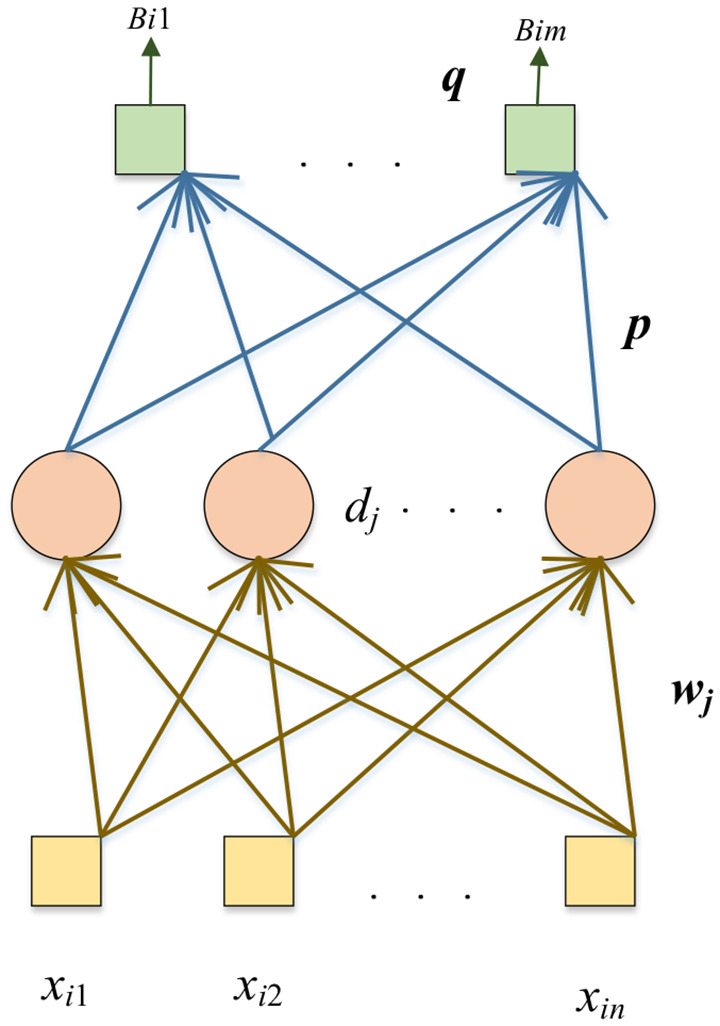
Structure of SNN.

Compared with the pre-trained DenseNet, randomized neural networks (RNNs) have a much shorter training time. In the DSNN, we replace the end five layers of the fine-tuned DenseNet with the RNN: the Schmidt neural network (SNN; Schmidt et al., 1992). The SNN is trained by extracted features *n* from FC128. The structure of the SNN is shown in Figure 4.

The yellow box is the input, the pink circle represents the hidden nodes, and the green box shows the output. Given *N* samples and dataset with the *i*-th sample as (***x_i_, y_i_***):


(4) $$x_{i}={\left(x_{i1},...,x_{in}\right)}^{T}\in R^{n},\;i=1,...,N,$$

(5) $$y_{i}={\left(y_{i1},...,y_{in}\right)}^{T}\in R^{m},\;i=1,...,N,$$

where *n* is the input dimension, *m* is the output dimension.

The training algorithm of SNN is as follows. The weights vector (***w_j_***) connects the *j*-th hidden node with input nodes, *d_j_* is the bias of the *j*-th hidden node. The weights vector (***w_j_***) and the bias (*d_j_*) are assigned with random values and will remain unchanged during the training process. The output matrix of the hidden layer with *V* hidden nodes is calculated as follows:


(6) $${\text{A}}_{\text{SNN}}=\sum_{j\;=\;1}^{V}s(w_{j}x_{i}+d_{j}),i=1,...,N,$$

where the sigmoid function is represented as *s*(). Then we use pseudo-inverse to calculate the final output weights (***P***):


(7) $$(P,q)={\text{A}}_{\text{SNN}}^{\text{†}}\text{Y,}$$

where the output biases of SNN are ***q***, ${\text{A}}_{\text{SNN}}^{\text{†}}$ is the pseudo-inverse matrix of **A_SNN_**, and **Y** = (**(*y_1_,....,y_N_*)^T^** is the ground-truth label matrix of the dataset.

**Figure 5:**
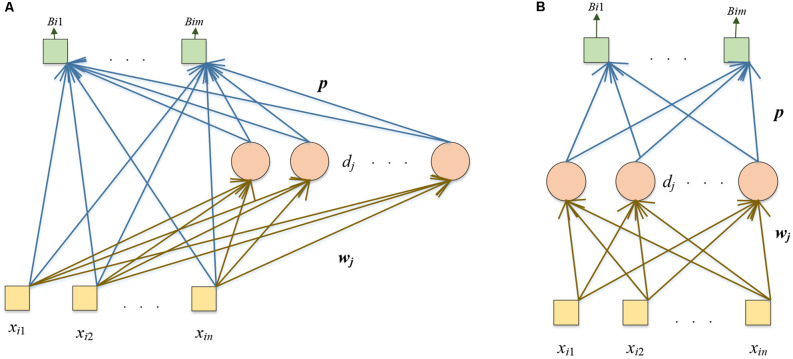
Thestructures of **(A)** RVFL and **(B)** ELM.

We propose two other models: DRVFL and DELM. The backbone of the two other proposed models is the pre-trained DenseNet. We modify the pre-trained DenseNet in the two proposed models as the “modifications of the pre-trained DenseNet” in the DSNN. We replace the softmax and classification layer of the pre-trained DenseNet with six layers: FC128, ReLU, BN, FC2, softmax, and classification layer. We fine-tune the modified DensNet by the training set. In the DRVFL, we select RVFL (Pao et al., 1994) to substitute the last five layers of the fine-tuned DenseNet. The structure of RVFL is shown in Figure 5A. In the DELM, ELM (Huang et al., 2006) is chosen to replace the last five layers of the fine-tuned DenseNet. The structure of ELM is shown in Figure 5B. ELM and RVFL are two types of RNNs.

The yellow box represents the input, the pink circle denotes the hidden nodes, and the green box shows the output. The difference between these two RNNs is that there are shortcut connections from the input to the output in RVFL. The calculation steps are similar:


(8) $$x_{i}={\left(x_{i1},...,x_{in}\right)}^{T}\in R^{n},\;i=1,...,N,$$

(9) $$y_{i}={\left(y_{i1},...,y_{in}\right)}^{T}\in R^{m},\;i=1,...,N,$$

Given *N* samples and dataset with the *i*-th sample as (***x_i_, y_i_***)

where *n* is the input dimension, *m* is the output dimension. The training steps of these two RNNs are as follows:

**Step 1:** ***w_j_*** is the weight vector, which connects the input nodes with the *j*-th hidden node. The bias of the *j*-th hidden node is represented as *d_j_*. We randomly assign ***w_j_*** and *d_j_* with values. These values will not change in training.

**Step 2:** The hidden layer’s output matrix is calculated as:

For RVFL:


(10) $${\text{A}}_{\text{RVFL}}=\text{concat (X,K),}$$

where **X** = **(*x_1_,....,x_N_*)^T^** denotes the input matrix. The **K** is calculated as follows:


(11) $${\text{K}}_{\text{RVFL}}=\sum_{j=1}^{V}s(w_{j}x_{i}+d_{j}),i=1,...,N,$$

where *V* is the number of the hidden nodes in the hidden layer, *s*() represents the sigmoid function.

For ELM:


(12) $${\text{A}}_{\text{ELM}}=\sum_{j=1}^{V}s(w_{j}x_{i}+d_{j}),i=1,...,N,$$

**Step 3**: The output weights (***p***): can be calculated by pseudo-inverse:

For RVFL:


(13) $$p={\text{A}}_{\text{RVFL}}^{\text{†}}\text{Y,}$$

where ${\text{A}}_{\text{RVFL}}^{\text{†}}$ is the pseudo-inverse matrix of **A_RVFL_**, and **Y** = (*y_1_,...,y_N_*)^T^ is the ground-truth label matrix of the dataset.

For ELM:


(14) $$p={\text{A}}_{\text{ELM}}^{\text{†}}\text{Y,}$$

where ${\text{A}}_{\text{ELM}}^{\text{†}}$ is the pseudo-inverse matrix of **A_ELM_**.

The backbone of these other two proposed models in this article is the same. The difference is that DRVFL chooses RVFL as its classifier, and DELM selects ELM as its classifier.

### Evaluation

We define the unhealthy brain as the positive and the healthy brain as the negative. Five indicators are chosen to verify our model: accuracy (Acc), sensitivity (Sen), specificity (Spe), precision (Pre), and F1-score (F1), respectively. Their formulas are shown below:


(15) $$\{\begin{array}{c} \text{Acc = }\frac{\text{TP + TN}}{\text{TP + TN + FP + FN}}\\ \text{Sen = }\frac{\text{TP}}{\text{TP + FN}}\\ \text{Spe = }\frac{\text{TN}}{\text{TN + FP}}\\ \text{PRe = }\frac{\text{TP}}{\text{TP + FP}}\\ \text{F1 = }\frac{\text{2×TP}}{\text{2TP + FP + FN}} \end{array}$$

where the definitions of TP, FN, FP, and TN are the true positive, false negative, false positive, and true negative, respectively.

## Results and Discussions

### Experiment Settings

We modify the hyper-parameter settings of the proposed DSNN. The max-epoch is set to 4 for reducing overfitting problems. We set our mini-batch size to 10 because the dataset is relatively small. According to the experience, the learning rate is 10^−4^. A hyper-parameter we set in our model is the number of hidden nodes (*V*), which is set as 400 based on the input dimension. The hyper-parameter settings of our model are shown in Table 5.

**Table 5:** The hyper-parameter settings of the proposed DSNN.

Hyper-parameter	Value
Mini-batch size	10
Max-epoch	4
Learning rate	10^−4^
Number of the hidden nodes V	400

### Performances of the DSNN

**Figure 6:**
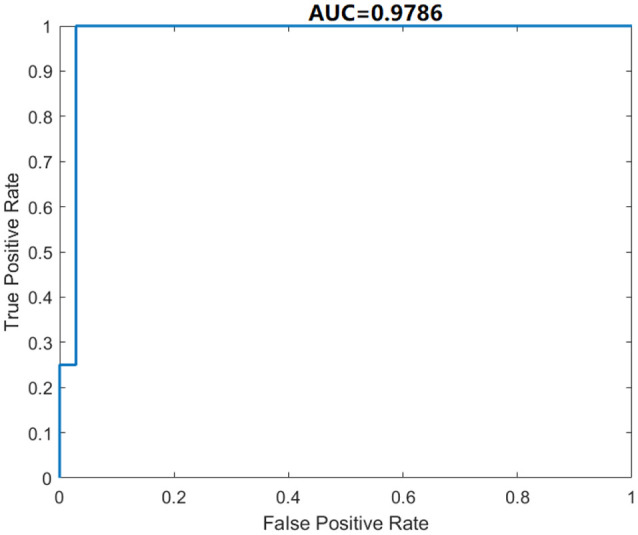
ROC curve of DSNN.

We use five-fold cross-validation to evaluate the proposed DSNN. The classification performance of our model is given in Table 6. The Acc, Sen, Spe, Pre, and F1 of the proposed DSNN are 98.46% ± 2.05% , 100.00% ± 0.00% , 85.00% ± 20.00% , 98.36% ± 2.17%, and 99.16% ± 1.11% , respectively. The results of DSNN are higher than 85%. Especially the sensitivity is 100%. The ROC curve is shown in Figure 6. The AUC value is 0.9786. It is an effective classifier when the AUC value is greater than 0.95. These results can be concluded that DSNN is an effective model to classify brain diseases.

**Table 6:** The classification performance based on five-fold cross-validation (unit: %).

Methods	Fold	Acc	Sen	Spe	Pre	F1
DSNN(Ours)	F 1	100.00	100.00	100.00	100.00	100.00
	F 2	100.00	100.00	100.00	100.00	100.00
	F 3	94.87	100.00	50.00	94.59	97.22
	F 4	100.00	100.00	100.00	100.00	100.00
	F 5	97.44	100.00	75.00	97.22	98.59
	Avr	**98.46**	**100.00**	**85.00**	**98.36**	**99.16**
	Std	±2.05	±0.00	±20.00	±2.17	±1.11
DRVFL(Ours)	F 1	100.00	100.00	100.00	100.00	100.00
	F 2	100.00	100.00	100.00	100.00	100.00
	F 3	89.74	100.00	0.00	89.74	94.59
	F 4	100.00	100.00	100.00	100.00	100.00
	F 5	97.44	100.00	75.00	97.22	98.59
	Avr	97.44	100.00	75.00	97.39	98.64
	Std	±3.97	±0.00	±38.73	±3.97	±2.10
DELM(Ours)	F 1	100.00	100.00	100.00	100.00	100.00
	F 2	100.00	100.00	100.00	100.00	100.00
	F 3	92.31	100.00	25.00	92.11	95.89
	F 4	100.00	100.00	100.00	100.00	100.00
	F 5	97.44	100.00	75.00	97.22	98.59
	Avr	97.95	100.00	80.00	97.87	98.90
	Std	±2.99	±0.00	±29.15	±3.07	±1.60
Fine-tuned DenseNet	F 1	87.50	86.11	100.00	100.00	92.54
	F 2	82.05	80.00	100.00	100.00	88.89
	F 3	89.74	88.57	100.00	100.00	93.94
	F 4	85.00	83.33	100.00	100.00	90.91
	F 5	79.49	77.14	100.00	100.00	87.10
	Avr	84.76	83.03	100.00	100.00	90.67
	Std	±3.67	±4.10	±0.00	±0.00	±2.46
AlexNet-SNN	F 1	89.74	100.00	0.00	89.74	94.59
	F 2	89.74	100.00	0.00	89.74	94.59
	F 3	90.00	97.22	25.00	92.11	94.59
	F 4	90.00	97.22	25.00	92.11	94.59
	F 5	89.74	97.14	25.00	91.89	94.44
	Avr	89.84	98.32	15.00	91.12	94.56
	Std	±0.13	±1.38	±12.25	±1.13	±0.06
ResNet-18-SNN	F 1	100.00	100.00	100.00	100.00	100.00
	F 2	97.50	100.00	75.00	97.30	98.63
	F 3	100.00	100.00	100.00	100.00	100.00
	F 4	94.87	100.00	50.00	94.59	97.22
	F 5	94.87	97.14	75.00	97.14	97.14
	Avr	97.45	99.43	80.00	97.81	98.60
	Std	±2.29	±1.14	±18.71	±2.03	±1.26
ResNet-50-SNN	F 1	95.00	94.44	100.00	100.00	97.14
	F 2	100.00	100.00	100.00	100.00	100.00
	F 3	97.44	97.14	100.00	100.00	98.55
	F 4	95.00	100.00	50.00	94.74	97.30
	F 5	100.00	100.00	100.00	100.00	100.00
	Avr	97.49	98.32	90.00	98.95	98.60
	Std	±2.24	±2.23	±20.00	±2.10	±1.24
VGG-SNN	F 1	97.50	100.00	75.00	97.30	98.63
	F 2	87.50	94.44	25.00	91.89	93.15
	F 3	94.87	97.14	75.00	97.14	97.14
	F 4	89.74	100.00	0.00	89.74	94.59
	F 5	87.18	88.57	75.00	96.88	92.54
	Avr	91.36	96.03	50.00	94.59	95.21
	Std	±4.12	±4.26	±31.62	±3.16	±2.33
Restricted DenseNet-SNN	F 1	94.87	94.29	100.00	100.00	97.06
	F 2	100.00	100.00	100.00	100.00	100.00
	F 3	97.37	97.06	100.00	100.00	98.51
	F 4	94.87	100.00	50.00	94.59	97.22
	F 5	100.00	100.00	100.00	100.00	100.00
	Avr	97.42	98.27	90.00	98.92	98.56
	Std	±2.09	±1.83	±1.97	±2.09	±1.69

The bold values are results of our proposed model.

### Comparison of Three Proposed Models

**Figure 7:**
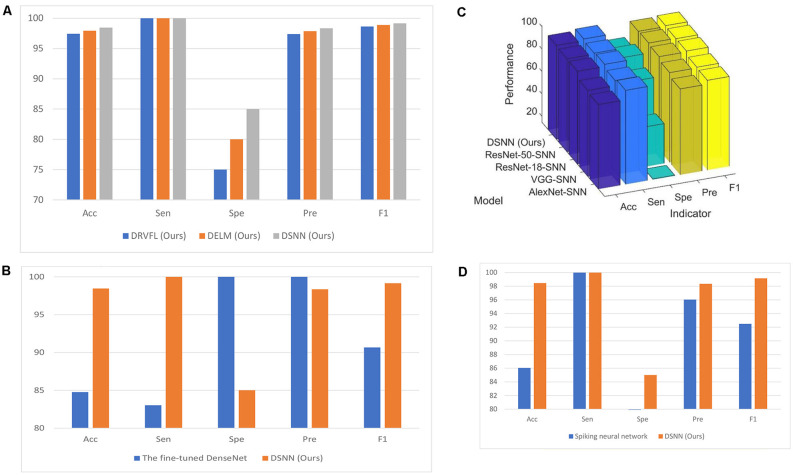
Modelcomparison. **(A)** Comparison of three proposed models (unit:%). **(B)** Comparison with the fine-tuned DenseNet (unit: %).**(C)** The classification performance of the proposed DSNN withdifferent backbones (unit: %). **(D)** Comparison with thespiking neural network (unit: %).

The classification performances of DRVFL and DELM based on the five-fold cross-validation are shown in Table 6. For a more explicit comparison, the comparison figure of the three proposed models is presented in Figure 7A. The proposed DSNN is 1.06% more accurate than the proposed DRVFL and 0.53% more accurate than the proposed DELM. The proposed DSNN gets the best performance among the three proposed models because there is an output bias in SNN.

### Comparison With the Fine-Tuned DenseNet

We compare the proposed DSNN with the fine-tuned DenseNet. The classification performance of the fine-tuned DenseNet is given in Table 6. The comparison figure of the proposed DSNN with the fine-tuned DenseNet is given in Figure 7B. It can be seen from Table 6 and Figure 7B that the accuracy of the proposed DSNN results is 13.97% greater than that of the fine-tuned DenseNet.

DenseNet has too many layers and parameters and is prone to meet overfitting problems because our dataset is relatively small. The structure of SNN is simple and has only three layers. What’s more, there are fewer parameters in SNN, which is not easy to produce overfitting problems. So, our method achieves better accuracy than fine-tuned DenseNet.

### Comparison of Different Backbones

We test the performance of the proposed DSNN with different backbones. These backbones are AlexNet, ResNet-18, ResNet-50, and VGG, respectively. The classification performances of the proposed DSNN with different backbones are shown in Table 6. For a clear comparison, the comparison of DSNN with different backbones is presented in Figure 7C.

DenseNet as the backbone model achieves the best results compared with other backbones. The reason is that DenseNet can reduce gradient vanishing problems better than other CNN models by establishing dense connectivity between all front and rear layers. There are too many parameters in VGG and AlexNet. There are 138M parameters for VGG and 61M parameters for AlexNet. However, there are only 20M parameters in DenseNet. More epoch is needed to converge for VGG and AlexNet. Nevertheless, to prevent overfitting problems, we set the max-epoch to 4. Therefore, DenseNet obtains better performance than VGG and AlexNet. Compared with ResNet, dense connections in the layers can provide more supervision information so that DenseNet can produce better classification performance. DenseNet has also shown its superiority in image learning in other studies, such as Ker et al. (2017), Zhang and Patel (2018), and Lundervold and Lundervold (2019).

### Comparison With Restricted DenseNet

We limit the number of connections in the DenseNet block. Each layer is only connected to the previous layer in the last block. The results are shown in Table 6. Except for the specificity (Spe) value, all other results are not as good as the results of the network we proposed. It is concluded that reducing some dense connections will not improve the classification performance.

### Comparison With Spiking Neural Network

We compare the proposed DSNN with the spiking neural network (Yaqoob and Wróbel, 2017). Although the brain inspires spiking and convolutional neural networks, there are still differences. The communication between neurons is completed in the spiking neural network by broadcasting the action sequence (Tavanaei et al., 2019). The final result of the spiking neural network is shown in Table 7. The comparison figure is given in Figure 7D. In conclusion, the performance of our model is better than the spiking neural network.

**Table 7:** The final result of the spiking neural network (unit: %).

Model	Acc	Sen	Spe	Pre	F1
Spiking neural network	86.05	100.00	0.00	96.05	92.50
**DSNN(Ours)**	**98.46**	**100.00**	**85.00**	**98.36**	**99.16**

The bold values are results of our proposed model.

### Explainability of the Proposed DSNN

**Figure 8:**
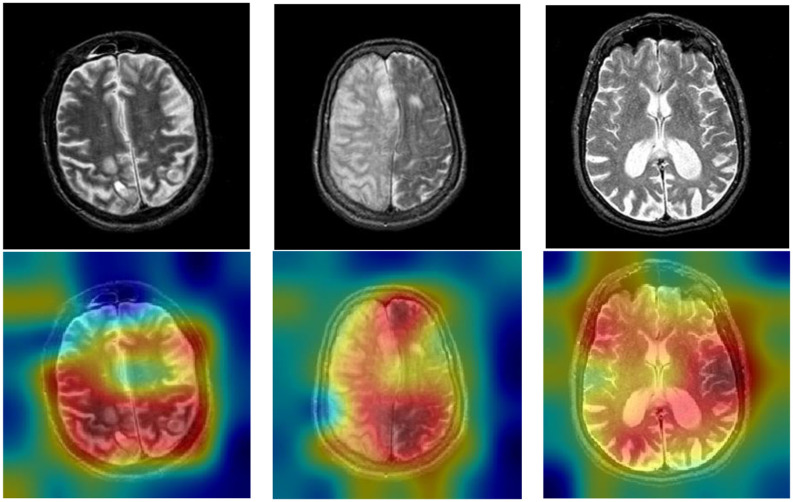
Explainability of the proposed DSNN.

It is significant to explain the DCNNs because it is difficult for researchers to figure out how DCNNs make predictions. We can visualize the attention of DCNNs by the Gradient-weighted class activation mapping (Grad-CAM). We present the raw images and heatmap images in Figure 8. The brain diseases are within the red region, which is the greatest attention in Grad-CAM.

The blue region is the lowest attention in Grad-CAM. Based on the Grad-CAM, we can conclude that DSNN can classify brain diseases in MRI. Also, some other studies have proven that the Grad-CAM efficiently visualizes the attention of DCNNs, such as Chattopadhay et al. (2018), Woo et al. (2018), Chen et al. (2020), and Panwar et al. (2020).

### Comparison With Other State-of-the-Art Methods

**Figure 9:**
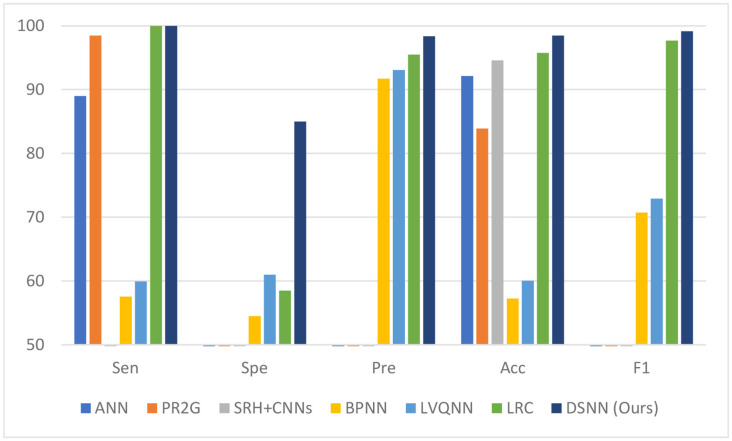
Comparison with other state-of-the-art methods (unit: %).

We compare the proposed DSNN with other state-of-the-art methods. These state-of-the-art methods are: ANN (Arunkumar et al., 2020), PR2G (Kalaiselvi et al., 2020), SRH + CNNs (Hollon et al., 2020), BPNN (Hemanth et al., 2011), LVQNN (Nayef et al., 2013), and LRC (Chen et al., 2017), respectively. The results are presented in Table 8. The comparison chart is given in Figure 9. The proposed DSNN gets the best performance among the list methods.

**Table 8:** Comparison with other state-of-the-art methods (unit: %).

Methods	Sen	Spe	Pre	Acc	F1
ANN (Arunkumar et al., 2020)	89.00	-	-	92.14	-
PR2G (Kalaiselvi et al., 2020)	98.46	-	-	83.90	-
SRH + CNNs (Hollon et al., 2020)	-	-	-	**94.6**	-
BPNN (Hemanth et al., 2011)	57.54	54.50	91.71	57.23	70.72
LVQNN (Nayef et al., 2013)	59.94	61.00	93.08	60.05	72.92
LRC (Chen et al., 2017)	100.00	58.50	95.47	95.74	97.68
DSNN (Ours)	**100.00**	**85.00**	**98.36**	**98.46**	**99.16**

*Bold means the best results, - means not available*.

Our model is an effective method to classify brain diseases based on the comparison results. The proposed DSNN can achieve these good results because deep learning is used to extract features, and SNN is used for classification.

## Conclusion

Three novel models are proposed to automatically classify brain diseases in this article. The proposed models are DSNN, DRVFL, and DELM. The DSNN gets the best performance among the three proposed models in terms of classification performance. The backbone of the proposed DSNN is the pre-trained DenseNet. We modify the pre-trained DenseNet. Then, the modified DenseNet is fine-tuned on the dataset. The last five layers within the fine-tuned DenseNet are substituted by the Schmidt neural network (SNN). In the proposed DSNN, the fine-tuned DenseNet plays the role of feature extraction. The extracted features train the SNN. We evaluate the proposed DSNN by using five-fold cross-validation. The accuracy, sensitivity, specificity, precision, and F1-score of the proposed DSNN on the test set are 98.46% ± 2.05%, 100.00% ± 0.00%, 85.00% ± 20.00%, 98.36% ± 2.17%, and 99.16% ± 1.11%, respectively. The proposed DSNN is compared with other state-of-the-art methods and obtains the best results among the list methods. Our model obtaining the best performance can conclude that DSNN is an effective model for classifying brain diseases.

Although the proposed model gets good results, this article still has some shortcomings. (1) The dataset is relatively small. (2) We divide the datasets into two categories. However, there are many kinds of brain diseases.

We will collect more data to test the proposed model in the future. Then, we will try to classify multiple brain diseases. What’s more, we will do more research on brain segmentation. We will try more new deep learning methods, such as VIT, attention learning, etc.

## Data Availability Statement

Publicly available datasets were analyzed in this study. This data can be found here: https://www.med.harvard.edu/aanlib.

## Author Contributions

ZZ: conceptualization, software, data curation, writing—original draft, writing—review and editing, visualization. SL: conceptualization, software, data curation, writing—review and editing. S-HW: methodology, software, validation, investigation, resources, writing—review and editing, supervision, and funding acquisition. JG: methodology, validation, formal analysis, resources, writing—original draft, writing—review and editing, supervision. Y-DZ: methodology, formal analysis, investigation, data curation, writing—original draft, writing—review and editing, visualization, supervision, project administration, and funding acquisition.

## Conflict of Interest

The authors declare that the research was conducted in the absence of any commercial or financial relationships that could be construed as a potential conflict of interest.

## Publisher’s Note

All claims expressed in this article are solely those of the authors and do not necessarily represent those of their affiliated organizations, or those of the publisher, the editors and the reviewers. Any product that may be evaluated in this article, or claim that may be made by its manufacturer, is not guaranteed or endorsed by the publisher.
